# Posttraumatic stress disorder and its risk factors among adolescent survivors three years after an 8.0 magnitude earthquake in China

**DOI:** 10.1186/1471-2458-14-1073

**Published:** 2014-10-15

**Authors:** Yali Tian, Thomas KS Wong, Jiping Li, Xiaolian Jiang

**Affiliations:** Department of Cardiovascular Surgery, West China Hospital, Sichuan University, 37 Guo Xue Xiang Street, Chengdu, Sichuan Province 610041 China; Tung Wah College, Kowloon, Hong Kong

## Abstract

**Background:**

Serious and long-lasting psychiatric consequences can be found in children and adolescents following earthquake, including the development of posttraumatic stress disorder (PTSD). Although researchers have been focused on PTSD recently, its prevalence and risk factors after a huge natural disaster are still unclear because of limited sample size. The purpose of this study is to explore the prevalence of posttraumatic stress disorder (PTSD) in adolescent survivors three years after the Wenchuan earthquake, describe PTSD symptoms, and to find out risk factors of PTSD.

**Methods:**

A total of 4,604 adolescents from three middle schools which located in earthquake-stricken areas were recruited in this study. Instruments included the demographic questionnaire, questionnaire about earthquake exposure, the Social Support Appraisal Scale (SSA), the Posttraumatic stress disorder Checklist-Civilian Version (PCL-C), and the structured clinical interview for DSM-IV Disorders (SCID).

**Results:**

The prevalence rate of PTSD was 5.7% (frequency: n = 261), and the most commonly occurring symptoms of PTSD were distress at reminders (64.5%), difficulty concentration (59.1%), and being easily startled (58.6%). Loss of houses and property, being injured, deaths of family members, and witness of death are positive risk factors of PTSD, and physical exercise and social support are negative risk factors of PTSD.

**Conclusions:**

Professional and effective interventions are needed to reduce the development of PTSD among adolescents after the Wenchuan earthquake, especially for these who lost their houses or property and lost their family members, witnessed death, and lacked of social support in the earthquake. Moreover, injured adolescents and adolescents who lacked of physical exercise also need intervention due to high risk.

## Background

At 14: 28 pm (local time) on 12^th^ May 2008, a devastating earthquake measuring 8.0 degree on the Richter scale struck the Wenchuan County, which is located in the center of Sichuan Province, China. There were almost 100,000 persons dead and around 370,000 people injured, and at least 14,000 schools were destroyed in the earthquake. Lots of adolescents lost their homes and family members, and suffered great physical and psychosocial damage.

Serious and long-lasting psychiatric consequences can be found in children and adolescents following earthquake, including the development of posttraumatic stress disorder (PTSD) [1–3]. PTSD is a series of chronic emotional responses to a traumatic event or situation involving severe environmental stress, with symptoms of re-experiencing, avoidance and hyper arousal.

It has been shown that earthquakes might cause PTSD, 4.5% to 95% children and adolescent who experienced the earthquake was reported to develop this disorder [1–11]. Many studies found that girls exposed to earthquakes are more likely to develop PTSD than boys [4–7]. Besides, earthquake experiences such as witness of injury and death [1], loss of house and property [6], injury of family members and/or relatives [7, 8], bury [8], death of family members [7, 8], and injury [6, 9] were risk factors to the development of PTSD in adolescent survivors. They postulated that at higher level of exposure to earthquake, the horrible memory in which adolescents injured, buried, or witnessed injury and death would be re-experienced in their lives; those adolescents were more likely to get PTSD. However, some study claimed that witness of injury and/or death [2], age [9], and gender [10] were not associated with PTSD symptoms in adolescents. Lack of social support after natural disaster and traumatic event was also found to be related to PTSD symptoms since insufficient supports might increase their fear, horror, and helplessness [10–12].

Previous study showed that the prevalence of PTSD was 45.5% in heavily damaged county and 9.4% in moderately damaged county in Wenchuan earthquake [10]. Liu and colleagues [8] also found that the incidence of PTSD was 54.3% in 118 wounded children and adolescents one month after the Wenchuan earthquake, and risk factors were being buried in the earthquake and injury of parents and relatives. Another research studied in three months after Wenchuan earthquake did not report the prevalence of PTSD; however, they found the high severity of PTSD symptoms among adolescents [12].

PTSD can last a long time even many years later. Previous surveys reported that PTSD would not appear in a short time after stressors but a long time after that [5–7]. Goenjian and colleagues [7] studied posttraumatic stress symptoms among 218 children 18 months after the 1988 earthquake in Armenia. The rates of PTSD were 26.0%, 71.0%, and 95.0% in three cities located at increasing distances from the epicenter of the earthquake. Separation from family members, lack of employment and housing, crowded living conditions, loss of community services, shortage of food, gasoline, medical supplies, and destroyed buildings contributed to PTSD symptoms. Another study collected data about disorder symptoms 20 months after the 1999 Turkey earthquake and found that the prevalence of PTSD was 39% [13]. The average duration of PTSD was 19 years; indeed, the duration or onset of symptoms can be longer [14]. And scholars should pay more attention to the long-term effects of earthquakes on young people. Another interesting phenomenon is that the prevalence of PTSD in a short time after earthquake may well be different from a long time after it. For most people, in front of a sudden trauma, unrealistic feeling would appear, then came more symptoms such as anxiety and painful memories, and avoidance of people and environment which was related to trauma. However, for next years, PTSD symptoms would decrease or disappear because of others’ interference, help, and support. Someone has shown that the prevalence rate of PTSD in the first month after Turky earthquake was twice as much as that in the thirteenth month [11].

These findings from literatures have important implications for preventing and treating stress disorders in adolescents after earthquakes. For the service provider, educations and interventions could be used to help adolescent survivors to deal with PTSD. Although researchers have been focused on PTSD recently, its prevalence and risk factors after a huge natural disaster are still unclear because of limited sample size. To our knowledge, only a few studies reported the rate of PTSD in the Wenchuan earthquake, with the absence of description of PTSD in adolescents. A survey with a large sample was required to be undertaken to find long-lasting mental influence of earthquake in adolescent survivors. We hypothesize that the prevalence of PTSD is not low and PTSD symptoms are serious in adolescent survivors three years after the Wenchuan earthquake, and risk factors such as physical exercise, earthquake exposure, and social support are associated with PTSD. So, the purposes of this study were: 1) to explore the prevalence of PTSD and describe PTSD symptoms in adolescent survivors three years after the Wenchuan earthquake; and 2) to find out associated risk factors of PTSD among adolescent survivors.

## Methods

### Ethical considerations and participant selection

Ethical approval was obtained from Sichuan University. In addition, informed consent was obtained from each adolescent and their guardians. They were assured that anonymity and confidentiality would be guaranteed and the right to withdraw from the study at any time. No pressure or inducement of any kind was applied to encourage adolescents to participate in the research.

Subjects were recruited from three most severely damaged middle schools among the 2008 Wenchuan earthquake, which located in three most severely earthquake-stricken counties in Sichuan Province. These schools were totally destroyed and students were studied in newly-built schools. Participants were considered for inclusion in this study if they were between 12 and 20 years old and experienced the Wenchuan earthquake. And the exclusive criteria included: 1) with problems in hearing, verbal communication, and vision; 2) refused to participate in this study. Finally, data were collected from 4805 adolescents, and data for 4604 respondents were completed and effective, businesslike for 95.8%.

### Participants

Among these adolescents, there were 2617 girls of an average age of 15.2 (SD = 1.8, range = 12–19) and 1987 boys of an average age of 15.1 (SD = 1.9, range = 12–19). Most of adolescents were the Han ethnic group (99.6%) and the only-child in their families (79.2%); more than half of them (57.0%) were senior middle school students; 72.5% of them took physical exercise frequently; and 26.2% of them had ever been student cadres. The data about earthquake exposure showed that 2.8% of them buried in this earthquake and 8.8% injured; some of them were with family members dead (7.7%) and handicapped (3.2%); over fifty percent (53.4%) lost their houses and property; 27.4%, 56.3%, and 29.9% of them witnessed burial, injury, and death in the earthquake respectively (Table 1 is about here).

**Table 1:** **Characteristics and earthquake exposure among adolescent survivors three years after the Wenchuan earthquake (n = 4604)**

Variable		N (%)
***Characteristics***		
Age (year)	Mean (SD)	15.0 (1.84)
Gender	Male	2617 (56.8)
	Female	1987 (43.2)
The ethnic group	The Han ethnic group	4584 (99.6)
	All others ^a^	20 (0.4)
Grade	Junior	1981 (43.0)
	Senior	2623 (57.0)
Only-child		3647 (79.2)
Had ever been a student cadre		1206 (26.2)
Took exercise frequently		3337 (72.5)
***Earthquake exposure experience***		
Being buried		131 (2.8)
Being injured		403 (8.8)
Being handicapped		56 (1.2)
Deaths, of		
family members		354 (7.7)
relatives or friends		1447 (31.4)
classmates or teachers		1272 (27.6)
Handicaps, of		
family members		146 (3.2)
relative or friends		548 (11.9)
classmates or teachers		988 (21.5)
Loss of house and property	Mildly	823 (17.9)
	Moderately	1323 (28.7)
	Severely	2458 (53.4)
Witness, of		1263 (27.4)
bury in the earthquake		3341 (72.6)
injury in the earthquake		2590 (56.3)
death in the earthquake		1378 (29.9)

^a^All others: include the Zang, Qiang, Hui ethnic group.

### Data collection

The research is a cross-sectional survey and took place three years after the Wenchuan earthquake. Before the assessments, all data collectors were called together to review the questionnaire and achieved agreement on the explanation of each item. Questionnaire included the demographic questionnaire, questionnaire about earthquake exposure, the Social Support Appraisal, the PTSD Checklist-Civilian Version (PCL-C), and the Structured Clinical Interview for DSM-IV Disorders (SCID). Based on scores of PCL-C, PTSD-positive cases (score of the PCL-C ≥50), partial PTSD cases (score of 38-50), and PTSD-negative cases (score of the PCL-C <38) were evaluated. Next, PTSD-positive cases were screened by psychiatrists using SCID to exclude the false positive cases. Finally, adolescents who were diagnosed as PTSD-positive by both the PCL-C and the SCID fitted into PTSD cases.

With the help of school staff who came from villages, townships and county-level cities, we identified the children who were present at school from Monday to Friday. Written informed consent was obtained from each subject and their guardians because they were adolescents and then carefully recorded by the interviewers after standard and complete descriptions of the study. Then each participant completed the questionnaire independently, and was presented a small gift upon its completion.

### Instruments

**Demographic data;** demographic variables included age, gender, the ethnic group, grade, being only-child or not, took physical exercise frequently (track and field sports three times or more in a week) or not.

**Earthquake exposure;** variables included buries, physical injuries, and handicaps in adolescent themselves, injuries and/or deaths in the family members, injuries and/or deaths in friends and teachers, witness of bury, injury, or death, and loss of house and property.

**The Social Support Appraisal Scale (SSA);** was revised by Professor Xin Ziqiang [15] into Chinese version according to Vaux’s Social Support Appraisal scale [16] to assess the level of social support among adolescent. The scale contains three subscales: supports from family members, friends and others. It includes respects, trusts, favors, cares and approval from family members, friends and others and the relationship between participants and their family members, friends and others. The scale consists of 20 items with a 5-point Likert format, 1 means “never”, 5 means “always”, total possible scores range from 20 to 100, and higher scores indicate higher support, with internal consistency of 0.91 for the scale and of 0.81 ~ 0.84 for three dimensions and a three-factor model was supported by the data ((χ^2^/df <5, NFI, NNFI and CFI above 0.95) in Xin’s study [15], and the internal consistency Cronbach’s alpha were 0.63, 0.65, 0.78 and 0.85 for supports from family members, friends, others and the whole scale respectively in the present study.

**The PTSD checklist-civilian version;** a 17-item self-report checklist of PTSD symptoms developed by the Behavioral Science Branch of American PTSD research center according to DSM-IV in 1994 for evaluating experience of ordinary people after trauma in normal life. The PCL-C is composed of three dimensions: the first dimension is re-experiencing of the traumatic events, which includes intrusive thoughts, bad dreams, fears recurrence, and distress at reminders; the second dimension is nominated avoidance of trauma-relevant stimuli and numbing of general responsiveness which includes feeling detached or estranged from others, and deriving markedly less pleasure from previously enjoyable activities; the third dimension includes symptoms such as irritability, hyper-arousal, and difficulties in sleep and concentration. Individuals would rate each item from 1 (“not at all”) to 5 (“extremely”) to indicate the degree to which they have been bothered by that particular symptom over the past month. Total possible scores range from 17 to 85. It is recommended that when the instrument is used as a continuous measure, a cut-off score of 50 is optimal for making the diagnosis of PTSD, and the score of 38-50 is diagnosed as partial PTSD [17]. This checklist has an excellent internal consistency (0.82-0.97), and excellent test–retest reliability over a 2 to 3 day period (0.96) was recorded. The checklist also correlates strongly with other measures of PTSD, such as the Mississippi Scale (0.93) and the Impact of Event Scale (0.90) [17, 18]. In the present study, Cronbach’s alpha for internal consistency were 0.82, 0.83, 0.83 and 0.91 for the dimensions of re-experiencing, avoidance and numbing, and increased arousal and the total scale respectively.

**The structured clinical interview for DSM-IV Disorders (SCID);** it was used to evaluate PTSD which is a clinician-administered diagnostic interview, corresponding to diagnostic criteria for PTSD in the DSM-IV [19]. The SCID was used by professional psychiatrists to evaluate the presence or absence of PTSD by information about the PTSD symptoms obtained from each participant. All individuals were asked questions about current (i.e., within the past month) and past (i.e., more than 1 month ago) disorders [20]. The assessment of PTSD included symptoms of PTSD resulting from the exposure to extreme traumata including re-experiencing the traumatic event, avoidance of stimuli associated with the trauma and numbing of general responsiveness, and symptoms of increased arousal. The post-traumatic stress disorder module of the SCID has been used with adolescents and the inter-rater kappa coefficient measuring reliability of interviewers was 0.72 for current and life-time PTSD [21].

### Data analysis

All statistical procedures were performed using the SPSS 13.0 software (SPSS Inc, Chicago, IL). Continuous data were described as means and standard deviations and as quartiles where appropriated. Multiple logistic regression analyses were performed to identify independent predictors of PTSD status. The level of significance was set at 0.05 (two-tailed).

## Results

### The prevalence of PTSD

After measured by the PCL-C and the SCID, the findings showed that 5.7% (n = 261) of the respondents were PTSD patients, 11.3% (n = 519) of them were partial PTSD patients.

### Posttraumatic stress disorder symptoms

**Figure 1:**
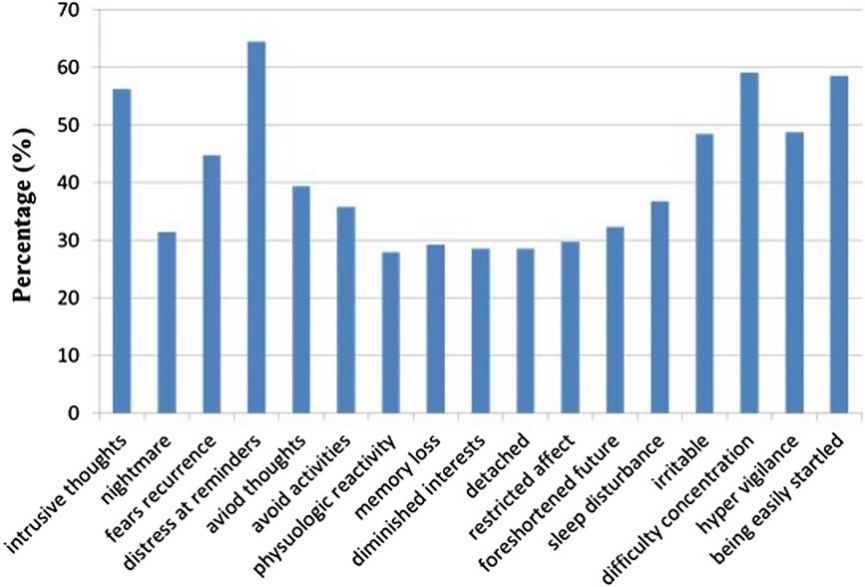
**The percentage of presence for each symptom item of PCL-C.**

The mean scores of PLC-C, re-experiencing, avoidance and hyper arousal were 28.11 ± 10.25 (range: 17-85), 8.37 ± 3.30 (range: 5-25), 10.59 ± 4.34 (range: 7-35), and 9.16 ± 3.91 (range: 5-25) respectively. Figure 1 showed the percentage of presence for each symptom item of PCL-C. The most commonly occurring symptoms were as follows: distress at reminders (64.5%), difficulty concentration (59.1%), being easily startled (58.6%), and intrusive thoughts (56.3%). The least commonly occurring symptoms were physiologic reactivity (28.0%), diminished interests (28.6%), memory loss (29.3%), and restricted affect (29.8%).

### Independent predictors of PTSD

Table 2 presents a multiple logistic regression analysis of the contribution of demographic features, earthquake exposure variables, and social support in predicting PTSD status. The six related factors entered as independent variables, loss of houses and property, being injured, deaths of family members, and witness of death are positive risk factors of PTSD, and physical exercise and social support are negative risk factors of PTSD.

**Table 2:** **Predictors of PTSD by logistic regression analysis among adolescent survivors (n = 4604)**

Variables	Odds	P	95%CI	
			Lower Upper	
Constant	2.046	.998		
Gender	1.265	.081	.971	1.648
Age	.961	.510	.854	1.082
Ethnic group	2.951	.468	.159	54.829
Grade	.782	.275	.504	1.215
Only-child status	.950	.747	.694	1.299
Physical exercise	.655	.003	.497	.863
Loss of house and property	1.569	<.001	1.273	1.934
Being injured	1.562	.035	1.033	2.362
Being buried	1.770	.079	.936	3.347
Being handicapped	1.156	.735	.499	2.677
Deaths of relatives	1.190	.238	.892	1.587
Deaths of family members	1.736	.005	1.178	2.558
Handicaps of relatives	1.337	.119	.928	1.927
Handicap of family members	1.200	.546	.664	2.167
Witness of death	1.338	.003	1.104	1.623
Witness of bury	1.094	.395	.890	1.344
Witness of injury	.962	.689	.795	1.164
Social support	.977	<.001	.965	.988

Note: CI = confidence interval.

## Discussion

To our knowledge, this was the first study to investigate the prevalence of PTSD in a large number of adolescent samples who experienced earthquakes in China. In this study, the prevalence of PTSD was 5.7%. It was lower than other earthquake studies among adolescents [1–3, 5–11], however, higher than the prevalence in Roussos’s [4] study after the 1999 earthquake in Ano Liosia. The Wenchuan earthquake was more powerful than other quakes, but the prevalence rate was lower than other reports except Roussos’s result. These differences may be referred to two factors. The first is performed time of assessment. Most of researches were studied in 3 months after stressors. But our study focused on a long term effect after quake. The second and the most important factor was the diagnosis measure of PTSD. The assessment of PTSD relies on two complementary sources: the patient’s self-report and the clinician’s assessment. Most studies used self-report questionnaires to screen for PTSD in major disasters [7–10]. However, the responses may have been over- or under- reported by the use of self-report questionnaires. So, in our study, after analysis of PCL-C, the PTSD-positive cases were assessed by psychiatrists using the SCID to exclude the false positive cases. Of course, the prevalence was lower because of further elimination and screening. The prevalence rates of PTSD diagnosed by structured clinical interview in other surveys were also not high [22–24], and one study even reported that it was only 1.1% far less than ours [24]. The joint use of self-report questionnaire and structured clinical interview also appeared in former studies [22, 23]. It can exclude the false positive cases as many as possible, further to make sure the accuracy of results.

Besides, there was 11.8% of partial PTSD as a result of the current study, which means that 17.5% (5.7% + 11.8%) of the adolescent survivors in this earthquake have had PTSD or had a great possibility of getting PTSD even three years after the Wenchuan earthquake, and an appropriate post-disaster mental health intervention program is needed to prevent the development of PTSD.

Previous study showed a high percentage of PTSD symptoms among adolescents after earthquake, especially distress at reminders (74.5%), being easily startled (68.8%), and intrusive thoughts (60.9%) and physiologic reactivity (66.7%) [24]. Another study of PTSD after a major earthquake showed that the most commonly occurring symptoms were difficult sleeping, being easily startled, and re-experiencing the event in a distressing way [25]. These results were similar with our study. Being suffered from intense fear, helplessness and horror, the adolescents had distressing recollections of the disaster with images and feelings of quakes, the percentage of distress at reminders is high (64.5%). Symptoms like difficulty concentration (59.1%), being easily startled (58.6%), and intrusive thoughts (56.3%) were also found in three years after earthquakes among adolescents in our study, which means that PTSD symptoms especially re-experiencing and increased arousal would persist for a long time even many years later.

Characteristic risk factors of PTSD symptoms in adolescents after disasters were widely studied. It has been shown that girls exposed to earthquakes are more likely to develop PTSD than boys [4–7]. However, our research did not find gender associated with PTSD, former study indicated that girls reacted more strongly to adversity than boys might be due to different coping style [12], and future study can focus on their relationship. Additionally, we found an interesting phenomenon that adolescents who lacked of proper physical exercise had a significantly higher risk on PTSD than those who took exercise frequently. This was consistent with prior finding that physical activity programs were associated with fewer symptoms of mental disease among adolescents [26]. Previous studies found that sports activity may increase adolescents’ self-esteem, and exercise programs could help to lessen anxiety and depression symptoms [27, 28]. Thus, education and sports interventions could be used to help adolescent survivors to prevent or deal with PTSD.

In this study, the physical injury caused by earthquake and deaths of family members were found as risk factors of PTSD. This is understandable and similar with most previous studies about adolescents after earthquakes [2, 9]. Similar with former studies [1, 10], the regression equation also showed that witness of death was significant predictors of the severity of PTSD symptoms. In our study, the earthquake occurred in the afternoon on a school day, and in some schools most of students were killed instantly in their classrooms. The horrible memory in which students witnessed schoolmates being crushed and wounded by collapsed and destroyed buildings would be re-experienced in several months or even several years later in their lives.

Similar with former studies [6], loss of house and property was one of major risk factors of PTSD in our study. According to Galea and colleague’s [29] studies, stressors after disaster include any loss of or damage to personal property (e.g., house, furniture, appliances, vehicles); and experiencing financial loss (e.g., lost job, fall in household income). With loss of houses and property, adolescents and their family members were faced with problems like persistent financial problems and difficulties with living arrangements even three years later. Adolescents would worry about the family’s situation and the future. The strong link between earthquake-related exposures and the evolution of PTSD indicates that earthquake exposures may increase symptomatology, the long-term course of posttraumatic stress disorder symptoms may depend on the persistent exposure to stressors [30].

This study also examined the predictive factors for PTSD. Social support was found to be major positive factor. Prior studies indicated that low levels of social support after the traumatic events were related to PTSD symptoms [10–12]. In our study, many adolescents lost their parents, relatives, and friends in the earthquake. Insufficient supports from their family members and friends might have increased their fear, horror, and helplessness. They didn’t know how to deal with the stress disorder caused by earthquakes without enough support. The high level of social support is useful in lessening the effects of trauma exposure and preventing the development of PTSD in survivors who suffered from stressful events [7]. Our findings highlight the positive role of sufficient social support.

### Limitations and strengths

Some limitations of this study are important to be highlighted. Firstly, most adolescents are the Han ethnic group and therefore the results may not be generalizable to other Chinese ethnic group populations. Secondly, data on important demographic variables such as history of psychiatric illness and family psychiatric illness were not collected. Finally, owing to the cross-sectional design, the present study only described the PTSD symptoms of adolescents three years after the earthquake. Longitudinal study is suggested to follow up with the changes.

Despite these limitations, this study is the first study reporting the prevalence of PTSD among adolescent survivors after the Wenchuan earthquake and its associated risk factors in a large sample (n = 4604) study. Current findings provided important information that education and mental health intervention are needed to help adolescent survivors to deal with PTSD after earthquakes.

## Conclusions

Professional and effective interventions are needed to reduce the development of PTSD among adolescents after the Wenchuan earthquake, especially for these who lost their houses or property and lost their family members, witnessed death, and lacked of social support in the earthquake. Moreover, injured adolescents and adolescents who lacked of proper physical exercise also need intervention due to high risk.

## References

[1] Ekşi A, Braun KL, Ertem VH, Peykerli G, Saydam R, Toparlak D, Alyanak B (2007). Risk factors for the development of PTSD and depression among child and adolescent victims following a 7.4 magnitude earthquake. Int J Psychiatry Clin Pract.

[2] Hsu CC, Chong MY, Yang PC, Yen CF (2002). Posttraumatic stress disorder among adolescent earthquake victims in Taiwan. J Am Acad Child Adolesc Psychiatry.

[3] Goenjian AK, Karayan I, Pynoos RS (1997). Outcome of psychotherapy among early adolescents after trauma. Am J Psychiatry.

[4] Roussos A, Goenjian AK, Steinberg AM, Sotiropoulou C, Kakaki M, Kabakos C, Karagianni S, Manouras V (2005). Posttraumatic stress and depressive reactions among children and adolescents after the 1999 earthquake in Ano Liosia, Greece. Am J Psychiatry.

[5] Giannopoulou I, Strouthos M, Smith P, Dikaiakou A, Galanopoulou V, Yule W (2006). Post-traumatic stress reactions of children and adolescents exposed to the Athens 1999 earthquake. Eur Psychiatry.

[6] Goenjian AK, Walling D, Steinberg AM, Karayan I, Najarian LM, Pynoos R (2005). A prospective study of posttraumatic stress and depressive reactions among treated and untreated adolescents 5 years after a catastrophic disaster. Am J Psychiatry.

[7] Goenjian AK, Pynoos RS, Steinberg AM, Najarian LM, Asarnow JR, Karayan I, Ghurabi M, Fairbanks LA (1995). Psychiatric comorbidity in children after the 1988 earthquake in Armenia. J Am Acad Child Adolesc Psychiatry.

[8] Liu KZ, Liang XM, Guo LT, Li Y, Li XR, Xin B, Huang MJ, Li YY (2010). The acute stress disorder in the paediatric surgical children and adolescents injured in the Wenchuan earthquake of China. Steess Health.

[9] Kolaitis G, Kotsopoulos J, Tsiantis J, Haritaki S, Rigizou F, Zacharaki L, Riga E, Augoustatou A, Bimbou A, Kanari N (2003). Posttraumatic stress reactions among children following the Athens earthquake of September 1999. Eur Child Adolesc Psychiatry.

[10] Kun P, Chen X, Han S, Gong X, Chen M, Zhang W, Yao L (2009). Prevalence of post-traumatic stress disorder in Sichuan Province, China after the 2008 Wenchuan earthquake. Public Health.

[11] Altindaga AT, Ozenb S, Sir A (2005). One-year follow-up study of posttraumatic stress disorder among earthquake survivors in Turkey. Compr Psychiatry.

[12] Zhang WQ, Jiang XL, Ho KW, Wu DM (2011). The presence of post-traumatic stress disorder symptoms in adolescents three months after an 8.0 magnitude earthquake in southwest China. J Clin Nurs.

[13] Salcıoglfu E, Basoglu M, Livanou M (2003). Long-term psychological outcome for non-treatment seeking earthquake survivors in Turkey. J Nerv Mental Disease.

[14] Zlotnick C, Warshaw M, Shea MT, Allsworth J, Pearlstein T, Keller MB (1999). Chronicity in posttraumatic stress disorder (PTSD) and predictors of course of comorbid PTSD in patients with anxiety disorders. J Trauma Stress.

[15] Xin ZQ, Chi LP, Geng LN, Zhao XM, Wong K (2007). The recension and application of The Social Support Appraisal. J Chinese Mental Health.

[16] Vaux A, Phillips J, Holly L (1986). The Social Support Appraisal (SS-A) scale: studies of reliability and validity. Am J Community Psychol.

[17] Weathers FW, Litz BT, Herman DS, Huska JA (1993). Paper Presented in the International Society for Traumatic Stress Studies. The PTSD Checklist (PCL): Reliability, Validity and Diagnostic Utility.

[18] Yang XY, Yang HA, Liu QG, Yang LZ (2007). The research on the reliability and validity of PCL-C and influence factors. J Chinese Health Psychol.

[19] American Psychiatric Association (1994). Diagnostic and Statistical Manual of Mental Disorders.

[20] First MB, Spitzer RL, Gibbon M, Williams JBW (1996). Structured Clinical Interview for DSM-IV Axis I Disorders (SCID-I Research Version 2.0).

[21] Pelcovitz D, Libov B, Mandel F, Kaplan S, Weinblatt M, Septimus A (1998). Posttraumatic stress disorder and family functioning in adolescent cancer. J Trauma Stress.

[22] Bornoz JM, Alonso J, Girolamo G, Graaf R, Haro JM, Kovess-Masfety V, Lepine JP, Nachbaur G, Negre-Pages L, Vilagut G (2008). Main Traumatic Events in Europe: PTSD in the European Study of the Epidemiology of Mental Disorders Survey. J Trauma Stress.

[23] de Assis MA, de Mello MF, Scorza FA, Cadrobbi MP, Schooedl AF, da Silva SG, de Albuquerque M, da Silva AC, Arida RM (2008). Evaluation of physical activity habits in patients with posttraumatic stress disorder. Clinics.

[24] Zhao CZ, Li JF, Wong MS (2000). Prevalence and correlated factors of PTSD in adolescents 17 months after earthquake. J Chinese Psychiatry.

[25] Liao SC, Lee YJ, Liu SK (2000). Acute stress syndromes in patients at an emergency medical station after a major earthquake. Taiwanese J Psychiatry.

[26] Pastor Y, Balaguer I, Pons D (2003). Testing direct and indirect effects of sports participation on perceived health in Spanish adolescents between 15 and 18 years of age. J Adoles.

[27] Manger TA, Motta RW (2005). The impact of an exercise program on posttraumatic stress disorder, anxiety, and depression. Int J Emerg Ment Health.

[28] Liu XC, Kurita H, Uchiyama M, Okawa W, Liu LQ, Ma DD (2000). Life events, locus of control and behavioral problems among Chinese adolescents. J Clin Psychol.

[29] Cerda M, Bordelois PM, Galea S, Norris F, Tracy M, Koenen KC (2013). The course of posttraumatic stress symptoms and functional impairment following a disaster: what is the lasting influence of acute versus ongoing traumatic events and stressors?. Soc Psychiatry Psychiatr Epidemiol.

[30] Galea S, Tracy M, Norris F, Coffey SF (2008). Financial and social circumstances and the incidence and course of PTSD in Mississippi during the first 2 years after Hurricane Katrina. J Trauma Stress.

[CR31] The pre-publication history for this paper can be accessed here: http://www.biomedcentral.com/1471-2458/14/1073/prepub

